# Technical Performance Score: A Robust Predictor of Morbidity Following the Norwood Procedure at a Developing Country Institution

**DOI:** 10.21470/1678-9741-2024-0442

**Published:** 2025-06-11

**Authors:** Davi Freitas Tenório, Leonardo Augusto Miana, João Guilherme Vidal Meyer, Eric Shih Katsuyama, Christian Ken Fukunaga, Aida Luiza Ribeiro Turquetto, Luiza Patrick Amato, Marcelo Biscegli Jatene, Fabio B. Jatene

**Affiliations:** 1 Division of Pediatric Cardiovascular Surgery, Instituto do Coração (InCor), Hospital das Clínicas da Faculdade de Medicina da Universidade de São Paulo, São Paulo, São Paulo, Brazil; 2 Division of Surgery, University of Texas at Austin, Austin, Texas, United States of America; 3 Department of Medicine, Centro Universitário Faculdade de Medicina do ABC, São Paulo, São Paulo, Brazil

**Keywords:** Hypoplastic Left Heart Syndrome, Norwood Procedure, Surgery For Congenital Heart Diseases, Reference Standards, Hospital Mortality

## Abstract

**Introduction:**

The Norwood operation has transformed the approach to hypoplastic left heart syndrome and its variants. Given the complexity of this procedure, postoperative residual injuries are prevalent.

**Objective:**

To evaluate the impact of significant residual injuries on clinical outcomes and mortality in Norwood procedure patients at a high-volume tertiary center in a developing nation using the technical performance score (TPS).

**Methods:**

This single-center, retrospective study included patients who underwent the Norwood procedure between December 2018 and February 2023. Data on demographics, echocardiograms, complications, intensive care unit stay, and mortality were collected. Logistic regression and linear analyses assessed the impact of TPS on outcomes.

**Results:**

Of 69 patients, nine (13%) were excluded due to incomplete echocardiographic data, leaving 60 (87%) for TPS classification. Among them, 28 (47%) were male. TPS classification was as follows: 40 (66%) in class 1 (excellent), five (8.3%) in class 2 (adequate), and 15 (25%) in class 3 (inadequate), indicating significant residual lesions or need for reintervention. The 30-day mortality rate was 21.6%, increasing to 41.6% before the next stage. In TPS class 3, 30-day mortality was 33% vs. 17% in classes 1 and 2 (P = 0.27). Interstage mortality was 60% in class 3 compared to 35% in other groups (P = 0.13). Major complications were significantly higher in TPS class 3 (93% vs. 55.5%, P = 0.04).

**Conclusion:**

TPS effectively predicts major complications post-Norwood and serves as a valuable tool for improving patient outcomes.

**Figure:**
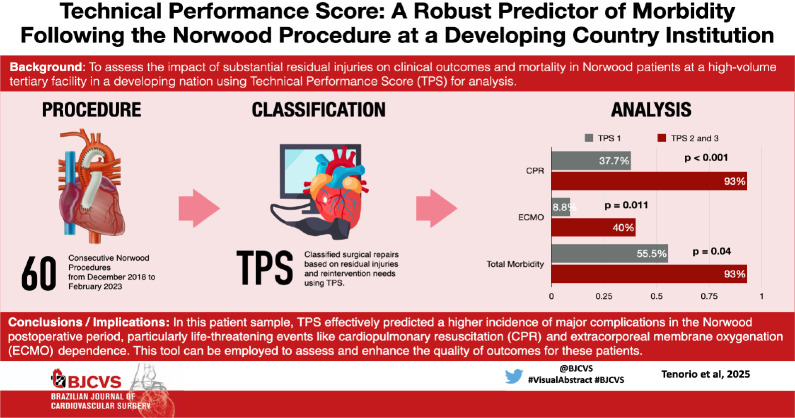

## INTRODUCTION

**Table:** 

Abbreviations, Acronyms & Symbols				
CPB	= Cardiopulmonary bypass		NW	= Norwood
CPR	= Cardiopulmonary resuscitation		PA	= Pulmonary artery
ECMO	= Extracorporeal membrane oxygenation		RACHS	= Risk Adjustment for Congenital Heart Surgery
HLHS	= Hypoplastic left heart syndrome		SACP	= Selective antegrade cerebral perfusion
ICU	= Intensive care unit		TCA	= Total circulatory arrest
IQR	= Interquartile ranges		TPS	= Technical Performance Score
LOS	= Length of stay		VIS	= Vasoactive-inotropic score

The Norwood (NW) procedure has revolutionized the treatment paradigm for hypoplastic left heart syndrome (HLHS) and its variants^[1]^. Before its establishment in the 1980s, this diagnosis was often considered a death sentence. Today, despite its inherent complexity, multiple institutions have achieved satisfactory outcomes in treating this syndrome and its variants^[2^-^4]^. However, despite advancements in surgical outcomes, there remains significant variability in surgical techniques and management approaches across different centers^[4^-^6]^.

Mortality associated with the NW procedure remains the highest among common congenital heart interventions, ranging from 5.7% to 21% in North America^[6]^. The largest published Brazilian cohort reports an early survival rate of 91.7%^[4]^; however, national data from the Departamento de Informática do Sistema Único de Saúde (or DATASUS)^[7]^ indicate mortality rates exceeding 60% in the surgical treatment of these patients. A comprehensive analysis spanning 35 countries revealed that the NW procedure is associated with the highest costs and the third-longest length of hospital stay among interventions for structural heart defects^[8]^.

Between hospital discharge following the NW procedure and completion of the second stage, 4% to 15% of patients experience a fatal outcome^[9]^. Moreover, it should be noted that, in addition to the inherent complexities of patients with HLHS and its variants, the NW procedure itself is technically challenging. It involves intricate repairs of extremely delicate tissues and structures with minimal diameters, often requiring hypothermia, selective cerebral perfusion, and, in some cases, circulatory arrest^[1^,^5^,^10]^.

For these reasons, the incidence of post-procedural residual injuries is significant^[11^-^13]^. Nevertheless, there is a consensus that optimal surgical repair, with minimal or no residual damage, leads to better patient outcomes^[12^-^14]^.

Once an acceptable level of residual lesions has been established, understanding the true impact of these echocardiographic findings on patient outcomes becomes crucial. To address this, Boston Children’s Hospital developed a quality assessment tool called the Technical Performance Score (TPS), which quantifies residual injuries based on echocardiographic findings and the need for reintervention^[15]^. This tool has been translated and validated into Portuguese by our group^[11]^.

TPS has been identified by some authors as an independent predictor of outcomes after the NW procedure^[12]^. Previous studies have shown that inadequate technical performance is associated with unfavorable outcomes, regardless of preoperative disease severity or case complexity^[16^,^17]^. The objective of the current study is to apply this score to NW procedures performed at our institution, evaluating the impact of significant residual injuries on unfavorable outcomes in our setting.

## METHODS

An observational, retrospective study was conducted using electronic medical records of all NW procedures performed consecutively at a single institution from December 2018 to April 2023. This study received approval from the institutional ethics committee (registration number 95807418.1.0000.0068), and due to the global initiative to enhance quality and surgical performance, informed consent was not required.

Demographic data were collected, and the complexity risk score was calculated using the Risk Adjustment for Congenital Heart Surgery (RACHS) 1 score, where the NW procedure is classified within the RACHS-6 stratum. Additionally, cardiopulmonary bypass and aortic clamping times were recorded, along with serum lactate levels at the conclusion of surgery. The levels of inotropes and vasopressors administered during the procedure were analyzed using the vasoactive-inotropic score (VIS).

TPS was assessed in accordance with previous studies^[12^,^15]^ and based on the translation and validation of the score conducted by our group^[18]^. The assessment incorporated the intraoperative course, the final pre-discharge echocardiogram, catheterization data, and clinical status at discharge.

A TPS was assigned for each pre-defined subcomponent of the NW procedure based on echocardiographic findings or reintervention data: class 1 for no residual lesions, class 2 for minor residual lesions, and class 3 for major residual lesions or reintervention before discharge. Reintervention was defined as any unplanned procedure performed during the initial hospitalization within the anatomical area addressed during the index procedure.

The evaluated subcomponents included proximal arch gradient, distal arch gradient, coronary perfusion, adequacy of atrial septectomy, neoaortic valve regurgitation, and flow in the modified Blalock-Taussig shunt or Sano shunt (right ventricle-pulmonary artery connection). Echocardiograms conducted at hospital discharge or prior to the first unplanned reintervention within the initial hospitalization were analyzed, as outlined in Table 1^[18]^.

**Table 1:** Classification of the Technical Performance Score for the Norwood procedure based on each assessed subcomponent of the procedure. Adapted from Miana et al., 2021^[18]^.

Subprocedures	1	2	3
Proximal arch reconstruction	No residual gradient or narrowing (peak gradient < 20 mmHg) No apparent narrowing by imaging or color Doppler jet width	Mild narrowing Mild residual gradient (peak gradient 20 - 40 mmHg) < 30% narrowing by imaging or color Doppler jet width	Reintervention Moderate to severe stenosis (peak gradient > 40 mmHg) > 30% narrowing by imaging or color Doppler jet width
Distal arch reconstruction	No residual gradient or narrowing (peak gradient < 20 mmHg) No apparent narrowing by imaging or color Doppler jet width	Mild narrowing Mild residual gradient (peak gradient 20 - 40 mmHg) < 30% narrowing by imaging or color Doppler jet width	Reintervention Moderate to severe stenosis (peak gradient > 40 mmHg) > 30% narrowing by imaging or color Doppler jet width
Coronary perfusion	Unobstructed flow into proximal coronary arteries	Unobstructed flow into proximal coronary arteries	Need for reintervention during initial hospital stay. Evidence for obstructed coronary flow (*e.g.*, echocardiogram, catheterization, electrocardiogram)
Atrial septectomy	No or trivial gradient, mean gradient < 2 mmHg	Mean gradient 3 - 4 mmHg (unless intended)	Need for reintervention
	(if restrictive atrial septum left on purpose accept higher gradient)		Mean gradient > 4 mmHg
Source of pulmonary blood flow			
a. Modified Blalock-Taussig shunt	Patent	Patent	Reintervention
			Partial or complete occlusion or distortion of the branches of the PA
b. Right ventricular-PA conduit	Patent	Patent	Reintervention
			Partial or complete occlusion or distortion of the branches of the PA

PA=pulmonary artery

The final TPS was assigned based on the highest score among the subcomponents: TPS 1 if all subcomponents were classified as class 1; TPS 2 if one or more subcomponents were classified as class 2, but none received a class 3; and TPS 3 if one or more subcomponents received a class 3 or if there was a reintervention for residual lesions (surgical or catheter-based) before discharge.

Operative mortality at 30 days was defined as death occurring in the hospital or within 30 days after the operation if the patient had been discharged before this period. Interstage mortality was defined as death occurring before the next scheduled procedure (Glenn or Fontan surgery).

As an exclusion criterion, operations where the TPS could not be calculated due to the unavailability of adequate postoperative echocardiographic data were excluded.

### Outcomes

The primary outcome was operative mortality, while secondary outcomes included morbidity, postoperative complications, and postoperative length of stay (LOS). Additional covariate variables included in the adjustment analyses were perioperative characteristics such as age, VIS, and serum lactate levels at the end of surgery.

Postoperative complications, morbidity, or severe adverse events were defined based on criteria established by the Society of Thoracic Surgeons Congenital Heart Surgery Database (or STS-CHSD)^[8^,^10]^. These included:

1. Cardiopulmonary arrest
2. Need for extracorporeal membrane oxygenation (ECMO)
3. Re-exploration due to hemodynamic instability
4. Re-exploration due to bleeding
5. Mediastinitis
6. Diaphragm plication
7. Stroke
8. Renal failure requiring dialysis

Reoperations or surgical and catheter-based reinterventions within the anatomical repair areas were not considered adverse events, as they are already accounted for in the TPS.

### Statistical Analysis

For statistical analysis, data were described using medians and interquartile ranges (IQR) of 25% and 75% for continuous variables, and absolute numbers with percentages for categorical variables.

To evaluate associations between categorical outcome variables (*e.g.*, mortality and postoperative complications) and explanatory variables, simple and multivariate logistic regression models were applied. For associations involving continuous outcome variables, simple and multivariate linear regression models were used.

A significance level of 0.05 was adopted for all tests, with two-tailed hypotheses considered. Data organization and statistical analyses were conducted using Google Sheets (Google), R (version 4.2.3), and IBM Corp. Released 2016, IBM SPSS Statistics for Windows, version 26.0, Armonk, NY: IBM Corp.

## RESULTS

Among the 69 total patients, nine (13%) were excluded from the analysis due to insufficient echocardiographic data, leaving 60 patients (87%) eligible for analysis and TPS categorization. Of these, 32 (53%) were female, and 28 (47%) were male.

Of the analyzed cases, 46 (76%) were diagnosed with HLHS, while 24% were classified as variants with univentricular physiology, including hypoplasia or atresia of the aorta and hypoplasia or interruption of the aortic arch. The median follow-up time was 6.4 months, with IQR of 2.2 months (25^th^ percentile) and 7.8 months (75^th^ percentile). Only one patient was lost to follow-up after discharge from the index procedure.

Among the 60 patients analyzed, 40 (66%) were classified as TPS class 1, five (8.3%) as TPS class 2, and 15 (25%) as TPS class 3.

In the group of surgeries classified as TPS class 3, 11 patients (73.3%) underwent unscheduled interventions before discharge due to significant residual lesions. Four patients (26.6%) were classified as TPS 3 due to the presence of significant residual lesions but did not require reintervention.

Of the total patients undergoing unplanned reintervention (n = 11):

• Four (36.3%) underwent percutaneous aortoplasty;
• One (9.0%) had a stent implanted in the Glenn shunt;
• Two (18.1%) underwent repair of the Sano shunt; and
• Nine (81.8%) required percutaneous treatment for pulmonary artery stenosis.

Among those treated for pulmonary artery stenosis, stent implantation was successful in eight cases (72.7%), while one case (9.0%) involved ballooning of the pulmonary artery alone.

The median perfusion time was 211 minutes (IQR: 188 - 273 minutes), with anoxia time of 88 minutes (IQR: 80 - 126 minutes) and selective antegrade cerebral perfusion time of 63 minutes (IQR: 47 - 70 minutes). Total circulatory arrest was employed in 38 patients (63%), with a median time of nine minutes (IQR: 4 - 20 minutes).

Patient and procedural characteristics are summarized in Table 2.

**Table 2:** Patients’ characteristics and surgical features.

Variables	Total (n = 60)	TPS 1 (n = 40)	TPS 2 (n = 5)	TPS 3 (n = 15)	*P*-value
Age at Norwood (n)	28 (4 - 153)	25.5 (3 - 164)	32 (3 - 73)	25 (5 - 96)	0.703
Male sex (%)	28 (47%)	21 (52%)	3 (60%)	4 (26%)	0.213
HLHS (%)	46 (76%)	33 (82%)	2 (40%)	11 (73%)	0.100
Surgical characteristics (n)					
Time on CPB (min)†	211 (188 - 273)	210 (185 - 270)	187 (167 - 190)	242 (207 - 309)	0.06
Clamping time (min)†	88 (80 - 126)	86.5 (79 - 112)	84 (76 - 131)	108 (87 - 165)	0.07
SACP (min)†	63 (47 - 70)	63 (52 - 74)	47 (38 - 47)	65 (62 - 67)	0.257
TCA (min)†	9 (4 - 20)	6 (2 - 16)	2 (1 - 4)	10 (7 - 29)	0.422
VIS final†	10 (10 - 13)	19 (9 - 14)	10 (10)	12 (9 - 14)	0.212
Final serum lactate (mg/dl)†	41 (23 - 69)	39 (22 - 68)	31 (23 - 35)	64 (39 - 84)	0.018
TPS (%)					
1	40 (66%)				
2	5 (8.3%)				
3	15 (25%)				

†mean or median

Nominal variables were assessed using the Chi-square test, and for continuous variables, analysis of variance was performed

CPB=cardiopulmonary bypass; HLHS=hypoplastic left heart syndrome; SACP=selective antegrade cerebral perfusion; TCA=total circulatory arrest; TPS=Technical Performance Score; VIS=vasoactive-inotropic score

### Primary Outcomes

Analyzing 30-day in-hospital mortality, 13 deaths were observed among the 60 cases, resulting in a 30-day mortality rate of 21.6%. During the entire interstage period analyzed, 25 deaths occurred, yielding an interstage mortality rate of 41.6%.

Stratification by TPS classification revealed the following:

• TPS class 1: eight deaths occurred within the first 30 postoperative days (20%). An additional six interstage deaths were observed, resulting in an interstage mortality rate of 35% for patients without residual lesions.
• TPS class 2: no deaths were recorded within the first 30 days; however, two interstage deaths occurred, resulting in a total mortality rate of 40% for this group.
• TPS class 3: five deaths were recorded within the first 30 postoperative days, corresponding to a 30-day mortality rate of 33.3%. An additional nine interstage deaths occurred, leading to an aggregated interstage mortality rate of 60% for this group.

Details of mortality outcomes are provided in Table 3.

**Table 3:** Interstage and 30-day mortality in the entire studied group, segmented according to the Technical Performance Score (TPS).

Variables	Total (n = 60)	TPS 1 (n = 40)	TPS 2 (n = 5)	TPS 3 (n = 15)
30-day mortality	21.6 % (13)	20% (8)	0% (0)	33% (5)
Interstage mortality	41.6% (25)	35% (14)	40% (2)	60% (9)

When statistical analysis was conducted to assess differences in 30-day mortality and interstage mortality among the groups, both univariate and multivariate regression analyses showed no significant differences in mortality between the groups. Additionally, the Levene test for equality of variances revealed no significant differences among TPS classes 1, 2, and 3.

**Fig. 1:**
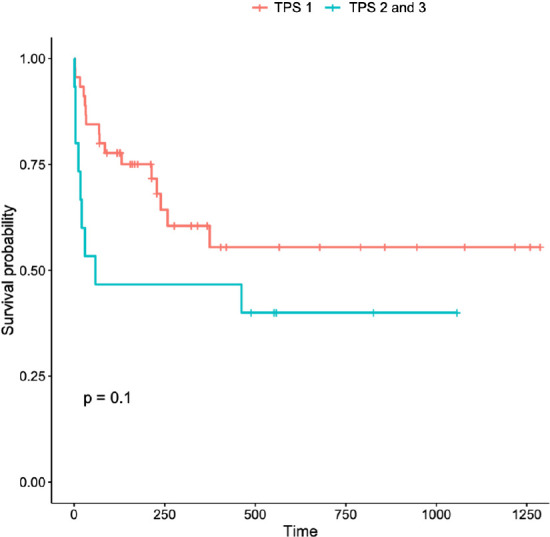
Kaplan-Meier curves for infants undergoing Norwood procedures. The red curve represents infants classified as Technical Performance Score (TPS) 1 and 2 combined (group A = adequate TPS), and the blue curve represents TPS 3 (group I = inadequate TPS). Time in days is on the x-axis, and the probability of survival is on the y-axis. The P-value was 0.1 for the difference in survival between the groups.

Due to the limited number of patients in TPS class 2, a comparative analysis of primary outcomes was performed by combining TPS classes 1 and 2 (surgeries without significant residual lesions) and comparing them with TPS class 3 (surgeries with technically inadequate outcomes). Figure 1 illustrates the comparative survival of these groups through Kaplan-Meier curves, with a *P*-value of 0.1. Thus, no significant differences were found in mortality and survival between these groups.

### Secondary Outcomes

When analyzing postoperative data, the median time to first extubation was nine days (IQR: 4 - 17), while the median LOS in the intensive care unit (ICU) was 24 days (IQR: 8 - 78), and the median length of hospital stay was 38 days (IQR: 17 - 129). Stratifying by TPS, the median time to first extubation was eight days (IQR: 3 - 15) in TPS class 1, seven days (IQR: 5 - 12) in TPS class 2, and 16 days (IQR: 9 - 19) in TPS class 3. The median length of ICU stay, stratified by TPS, was 21 days (IQR: 8 - 46) in TPS class 1, 73 days (IQR: 51 - 80) in TPS class 2, and 25 days (IQR: 14 - 123) in TPS class 3. Finally, the median length of hospital stay by TPS showed 33 days (IQR: 21 - 153) in TPS class 1, 84 days (IQR: 65 - 96) in TPS class 2, and 43 days (IQR: 14 - 113) in TPS class 3.

There were no significant differences between the medians of these times across the different TPS classes. The Kruskal-Wallis test showed *P*-values of 0.103 for time to first extubation, 0.594 for ICU LOS, and 0.898 for hospital LOS.

Regarding postoperative complications, 39 out of 60 cases (65%) presented some type of event. ECMO was required in 10 patients (16%). Reoperations were necessary in 19 cases (31%), with 10 (47%) due to bleeding and nine (53%) due to hemodynamic instability. Diaphragmatic plication was performed in seven cases (11.6%), and six patients (10%) experienced episodes of stroke. Renal insufficiency requiring dialysis was observed in 29 cases (48%). Finally, cardiopulmonary arrest was observed in 31 patients (51%).

When evaluating postoperative infections during the hospitalization period, 29 patients (48%) presented some type of infectious condition, including superficial surgical wound infections, and three patients (5%) developed mediastinitis. Table 4 summarizes the postoperative complications for the total group during the period.

**Table 4:** Summary of secondary outcomes.

Cases	n	Percentage
CPR	31	51.6%
ECMO	10	16.6%
Reoperation due to hemodynamic instability	9	15%
Reoperation for bleeding	10	16.6%
Mediastinitis	3	5%
Diaphragmatic plication	7	11%
Stroke (cerebrovascular accident)	6	10%
Acute kidney injury requiring dialysis	28	46%
Total morbidity	39	65%

CPR=cardiopulmonary resuscitation; ECMO=extracorporeal membrane oxygenation

When performing univariate logistic regression and comparing outcomes among the three groups, only total morbidity showed significance, with a *P*-value of 0.042 and an adjusted odds ratio of 18.56. The other variables were non-significant.

Due to the limited number of patients in TPS class 2, a comparative analysis of secondary outcomes was conducted between TPS classes 1 and 2 combined (absence of significant residual lesions) and TPS class 3 (technically unsatisfactory surgery). The Fisher’s exact test revealed a significant difference between the group with considered adequate TPS (TPS classes 1 and 2) and the group with considered inadequate TPS (TPS class 3) in total morbidity. This was primarily due to differences in cardiopulmonary resuscitation (CPR) and ECMO, which also achieved statistical significance individually.

Except for the three aforementioned morbidity comparisons, all other outcomes yielded non-significant results. Table 5 presents these outcomes, including total numbers, means, and *P*-values of the analyses, and stratifies complications according to TPS classes 1 and 2 *vs.* TPS class 3.

**Table 5:** Outcomes summary - associated TPS 1 and 2 (adequate TPS) e TPS 3 (inadequate).

TPS	Total		n	Mean	*P*-value
CPR	1 e 2	45	17	37.7%	< 0.001
	3	15	14	93%	
ECMO	1 e 2	45	4	8.8%	0.011
	3	15	6	40%	
Reoperation due to hemodynamic instability	1 e 2	45	5	11.1%	0.208
	3	15	4	27%	
Reoperation for bleeding	1 e 2	45	5	11.1%	0.102
	3	15	4	33%	
Mediastinitis	1 e 2	45	2	4.4%	0.585
	3	15	1	7%	
Diaphragmatic plication	1 e 2	45	5	11.1%	0.566
	3	15	2	13%	
Stroke (cerebrovascular accident)	1 e 2	45	3	6.6%	0.159
	3	15	3	20%	
Acute kidney injury requiring dialysis	1 e 2	45	20	44.4%	0.567
	3	15	8	53%	
Total morbidity	1 e 2	45	25	55.5%	0.04
	3	15	14	93%	
30-day mortality	1 e 2	45	8	17%	0.279
	3	15	14	33%	
Interstage mortality	1 e 2	45	16	35%	0.133
	3	15	9	60%	

*P*-value was calculated by Fisher's exact test

CPR=cardiopulmonary resuscitation; ECMO=extracorporeal membrane oxygenation; TPS=Technical Performance Score

## DISCUSSION

Previous studies have consistently demonstrated the negative impact of inadequate TPS on the morbidity and mortality of patients undergoing the NW procedure. These studies also highlight how appropriate surgical techniques can improve outcomes for patients with complex anatomies and suboptimal preoperative conditions^[14]^. Our findings further emphasize the significant influence of technical performance on patient morbidity.

The key takeaway from our study is the increased incidence of major complications among patients classified as TPS class 3 compared to those in TPS classes 1 and 2, underscoring the critical importance of technically accurate repairs. Specifically, 93% of patients in TPS class 3 experienced major complications, compared to 55.5% in TPS classes 1 and 2 - a statistically significant difference (*P* = 0.04). This result supports the use of TPS classification as a predictive marker for complication risk, with higher TPS classes correlating to an increased likelihood of adverse outcomes.

Patients with adequate TPS (classes 1 and 2) demonstrated significantly lower morbidity compared to those with inadequate TPS (class 3). Key contributors to this disparity included differences in CPR events and the need for ECMO, both of which showed individual statistical significance. These findings highlight the importance of achieving an adequate TPS to mitigate the risk of severe postoperative complications, particularly life-threatening events like CPR and ECMO dependence.

Similar observations have been previously reported in the literature, where inadequate TPS in congenital heart surgery was associated with a higher incidence of major adverse events. Nathan et al.^[13^,^14]^, for example, demonstrated through multivariate analysis that inadequate TPS, even after adjusting for confounding variables, was strongly associated with adverse outcomes, with an odds ratio of 6.9 (95% confidence interval, 4.1-11.7; *P* < 0.001) compared to optimal TPS^[13]^. Our study, however, is among the first ones to specifically correlate major adverse events with TPS class 2 (adequate) and TPS class 3 (inadequate) in the context of the NW procedure.

When comparing our TPS stratification results to those of the Boston Group, we observed a higher proportion of patients in TPS class 1 (66% *vs.* 58%) and TPS class 3 (25% *vs.* 16%), but a notably lower incidence of TPS class 2 (8.3% *vs.* 22%)^[12]^.

While our study did not have sufficient power to demonstrate a statistically significant influence of TPS on mortality, Kaplan-Meier survival curves (Figure 1) revealed a greater impact of adequate TPS on the immediate postoperative period. Over time, survival differences between groups stabilized following interventions to address residual lesions and progression to subsequent surgical stages. This trend suggests that the risk of mortality is particularly concentrated in the early postoperative period, a critical "window of vulnerability" when residual lesions or complications exert significant physiological stress on these high-risk neonates.

Addressing residual lesions through timely interventions - such as intraoperative echocardiography or early postoperative imaging with computed tomography angiography or cardiac catheterization - facilitated quicker reinterventions. These efforts likely helped shift some class 3 patients toward survival trajectories more aligned with classes 1 and 2, as previously suggested in the literature^[12^,^19]^. This approach was instrumental in improving surgical outcomes in our cohort, particularly in the subgroup of patients with HLHS.

The use of bridge therapies, such as ECMO, also played a crucial role in improving outcomes for patients in TPS class 3. This strategy provided time for identifying and addressing residual lesions, enabling successful weaning from ECMO and stabilizing physiology until the next stage of treatment.

### Limitations

As a retrospective study, this research is subject to inherent limitations, including the potential for loss to follow-up and missing data. However, these issues were minimized by the necessity for patients to undergo multiple staged procedures, ensuring continued monitoring over time.

While we did not observe a statistically significant relationship between TPS and mortality, the variable CPR was statistically significant among comorbidities. This discrepancy could be attributed to the study's sample size, as smaller samples are more likely to yield significant associations for high-frequency events like CPR or ECMO use, while rarer outcomes such as mortality may not achieve statistical significance.

It is also important to note that this study was conducted at a single center, which may limit the generalizability of its findings to other healthcare settings. Furthermore, patient categorization regarding TPS was based on echocardiograms performed at different time points during the initial hospitalization, which may have introduced variability in the results.

## CONCLUSION

In this patient sample, the TPS was able to predict a higher incidence of major postoperative complications in NW procedures. This tool can be used as an assessment instrument to enhance the quality of outcomes for these patients. As better results are achieved in mortality rates, postoperative morbidity becomes the next challenge, which, as an initial step to overcome, requires appropriate surgical performances.
